# The challenges faced by early career international medical graduates in general practice and opportunities for supporting them: a rapid review

**DOI:** 10.3399/BJGPO.2023.0012

**Published:** 2023-08-23

**Authors:** Alexandra Jager, Michael Harris, Rohini Terry

**Affiliations:** 1 University of Exeter Medical School, Exeter, UK; 2 Nuffield Department of Primary Care Health Sciences, University of Oxford, Oxford, UK; 3 Institute of Primary Health Care, University of Bern, Bern, Switzerland

**Keywords:** qualitative research, systematic reviews, general practice, primary health care

## Abstract

**Background:**

British general practice is facing a workforce crisis against a backdrop of an ageing population experiencing increasingly complex health challenges. The NHS must increase the supply of GPs, including international medical graduate (IMG) GPs, by increasing recruitment and retention. IMG GPs face distinct challenges during training and their early careers. Understanding these challenges, as well as the help and support offered to early career IMG GPs, is crucial to building and sustaining the general practice workforce.

**Aim:**

To understand the challenges facing early career IMG GPs and the help and support they can access.

**Design & setting:**

Rapid review of studies and grey literature on UK-based IMG GPs.

**Method:**

Six databases were searched. Four websites were searched to find grey literature. Titles and abstracts were screened according to inclusion and exclusion criteria, followed by the full study where applicable. The included studies were analysed using a thematic synthesis approach to identify the challenges faced by early career IMG GPs, as well as the help and support available.

**Results:**

The database search yielded 234 studies, with 38 additional studies identified via other methods. Twenty-one studies were included in the synthesis. Seven challenges were identified, as well as a range of help and support available. Early career IMG GPs face a range of psychological, social, and practical challenges, which may not be adequately addressed by the help and support currently offered by the NHS.

**Conclusion:**

Further research is required to understand the extent to which early career IMG GPs access the help and support offered, and if it adequately addresses the unique challenges they face.

## How this fits in

IMG GPs, while integral to fixing the NHS’s GP workforce crisis, face distinct challenges during their early careers. These include higher rates of complaints and disciplinary action than their UK-trained counterparts, suggesting they may require tailored help and support. This rapid review synthesised studies and grey literature on the challenges facing early career IMG GPs, as well as the help and support available to them. The study concluded that IMG GPs face unique practical, social, and psychological challenges that may not be adequately addressed by the help and support on offer. Policy recommendations have been provided to improve early career IMG GPs’ wellbeing and support their clinical practice.

## Introduction

As the UK's population has grown larger, older, more diverse, and more likely to seek professional medical help, the complexity and volume of work done by GPs over the past two decades has risen substantially.^1^ To address this, the NHS must increase the supply of GPs by increasing recruitment and improving retention.^1^

The NHS’s 2016 *General Practice Forward View* included plans to increase the GP workforce by 5000 by 2021, enabled in part by an international recruitment programme to attract up to 500 GPs from overseas.^2,3^ This target was later increased to 2000.^4^ However, in November 2021 there were 5% fewer fully trained whole-time equivalent GPs in England than 2015,^1^ and only 124 GPs recruited through the international recruitment programme were still practising in June 2022.^4^

IMG GPs perform worse than UK-graduate GPs in examinations during training,^5^ and are more likely to require training extensions.^6^ IMG GPs are more likely than UK-graduate GPs to leave the GP Register within 3 years of attaining a Certificate of Completion of Training (CCT)^7^or move abroad.^8^ The driving factors behind doctors (both IMGs and UK-trained doctors) leaving the UK include feeling undervalued professionally, and purported better working conditions and quality of life overseas.^8^ IMG GPs are more likely to receive complaints, with these complaints more likely to lead to sanctions or warnings.^9^

Consequently, while pivotal to addressing the NHS’s workforce crisis in general practice, IMG GPs face distinct challenges. Understanding these is crucial to identifying how the NHS can offer bespoke initiatives to build and sustain the GP workforce. This rapid review assessed the following:

1. What challenges do IMG GPs face early in their careers?
2. What help and support are available to early career IMG GPs, and how do these seek to facilitate their careers?

## Method

Six databases were searched in August 2022 (CINAHL [Cumulated Index to Nursing and Allied Health Literature], AMED [Allied and Complementary Medicine Database], Embase, MEDLINE, Web of Science, and Scopus), using a search strategy combining three terms: International Medical Graduates AND United Kingdom AND General Practice. Four websites were searched to identify grey literature (Supplementary Table S1). The results were imported into Covidence,^10^ deduplicated, and screened by one researcher, according to the inclusion and exclusion criteria (Table 1). A random sample of one in five of the studies was independently screened by a second researcher and the results of the screening compared. The inclusion and exclusion criteria were subsequently refined to ensure consistency during screening.

**Table 1.** Study inclusion and exclusion criteria

	Inclusion criteria	Exclusion criteria
Study population	The study is about IMGs in the UK or one or more of its constituent countries *or*The study is about IMGs in multiple countries including the UK (or one or more of its constituent countries) and the information or findings on UK-based IMGs are clearly differentiated from those on other IMGs (in this case only the portion of the study on UK-based IMGs will be read and coded)	The study is about IMGs in countries other than the UK *or*The study is about IMGs in multiple countries including the UK, but it is not possible to ascertain which information or findings are applicable to UK-based IMGs
Language	The study is written in English	
Study design	The study consists of the analysis of primary or secondary data, or is a descriptive or factual piece describing developments related to IMG GPs	The study is a conference abstract or presentationThe study is about the personal opinion of the author, for example in the form of a letter or transcribed lecture
Full text availability	The full text of the study is available	
Career stage	The study concerns the early career stage of an IMG GP, defined as IMG GPs who have been working as GPs in the UK for ≤10 years since obtaining one of the following: Certificate of Completion of Training (CCT) following a UK-based GP training programme Certificate of Eligibility for GP Registration (CEGPR): this route is available to the GP register for GPs not eligible for CCT because they have trained and worked as a GP outside the UK. This applies to overseas doctors with non-UK, non-European Economic Area (EEA) qualifications Alternatively, for IMG GPs who trained in the EEA and have a recognised European GP qualification, they must have begun working as GPs in the NHS within the past 10 years	The study concerns GP trainees or GPs in the later stages of their careers

IMG = international medical graduates

There is no universal definition of ‘early career clinicians,’^11^ but in the context of this review an early career IMG GP is defined as one who has worked as a GP in the UK for ≤10 years. The included studies did not typically state for exactly how long IMG GPs had been practising, so the researchers’ judgement decided whether the study met the inclusion criteria.

Studies were analysed in NVivo (version 12).^12^ Content relating to the challenges was coded and analysed thematically according to Thomas and Harden’s three-stage thematic synthesis approach, consisting of initial line-by-line coding, the organisation of coding into related high-level descriptive themes, and, where possible, the development of analytical themes.^13^ The final themes were both descriptive and analytical. Information relating to the help and support available was summarised descriptively.

## Results

**Figure 1.**
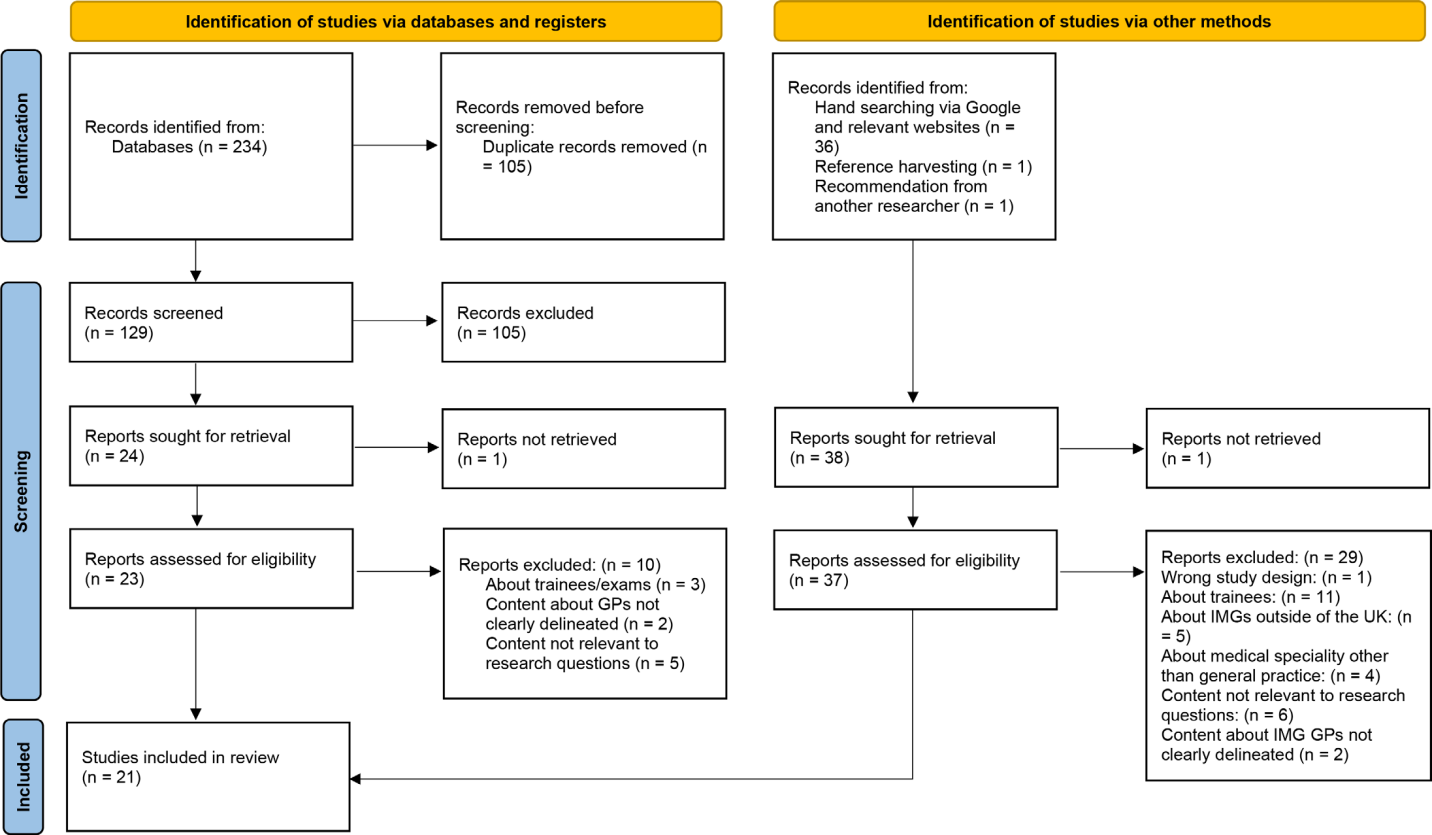
PRISMA flow chart. IMG = international medical graduate

The search results are summarised in the Preferred Reporting Items for Systematic Reviews and Meta-Analyses (PRISMA) diagram (Figure 1). Twenty-one studies were included (Supplementary Table S2), consisting of 15 pieces of peer-reviewed research^2,14–27^ and six pieces of grey literature,^7–28–30–30^ identified via database searching (*n* = 13), handsearching (*n* = 6), reference harvesting (*n* = 1), and recommendations (*n* = 1).

### Challenges facing early career IMG GPs

The following seven challenges were identified:

1. Complaints and disciplinary proceedings
2. Different GP consultation models
3. Discrimination and marginalisation
4. Geographical area of practice
5. Language and communication
6. Visas
7. Lack of NHS-specific knowledge

Sample supporting quotes are available in Supplementary Table S3.

#### Complaints and disciplinary proceedings

The relatively high rates of complaints (including from the public, other doctors, and employers) and disciplinary proceedings against IMG GPs were highlighted in two studies.^9,30^ Black and Minority Ethnic (BAME) IMG GPs were complained about more frequently than other ethnic groups; between 2012 and 2016, 25% of BAME IMG GPs received complaints, compared with 17% of their UK-graduate counterparts.^30^ Of doctors on the GP register in 2011–2015, both BAME and White IMG GPs were more likely to receive complaints, as well as sanctions or warnings, than their UK-graduate counterparts.^9^

#### Different GP consultation models

Three studies mentioned that some IMG GPs trained in countries with GP consultation models that could appear to be ‘*dominant*’ and ‘*doctor-centred*’ to their UK colleagues and patients, even if the IMG GPs were clinically competent and practising a consultation style appropriate to their native culture.^20,21,30^ The studies described the UK-model of doctor–patient relationships as more ‘*patient-centred*’^21^ and associated with less expected deference from patients, which IMG GPs may not have been adequately trained for in their home countries.^30^

#### Discrimination and marginalisation

The literature described incidences of IMG GPs experiencing discrimination and marginalisation related to race, ethnic group, or sex at the hands of patients and clinicians.^18,25,27,30^ In one study, the authors argued that IMG GPs face not only racism but also heterophobia, namely a fear and rejection of difference or ‘fear of the other,’ which can lead to marginalisation.^27^ South Asian female IMG GPs described suffering sexist discrimination from other IMGs.^27^ IMG GPs faced discrimination from White medical colleagues^25^ and others in the NHS.^30^ This could lead to exclusion from job opportunities, such as BAME IMG GPs being prevented from joining ‘White’ GP practices, although this discrimination was sometimes covert.^30^

Patients sometimes refused to be treated by an IMG GP on the basis of the GP’s ethnic group.^27^ A study of 395 White, native English adults concluded that they preferred UK-graduate GPs to those who graduated in Asia; participants preferred older GPs who had graduated in Asia to younger ones, possibly because they spoke better English and were more aware of cultural norms and customs.^18^

One study found that building social networks may be difficult owing to cultural reasons, and described the marginalisation of female migrant doctors and those who did not drink alcohol at events.^27^

#### Geographical area of practice

Seven studies noted that IMG GPs were more likely to work in poorer areas than UK-graduate GPs, with concomitant population health challenges.^8,15,19,25–30,30^ IMG GPs were more likely to work in deprived areas,^8,30^ South Asian GPs were more likely to work in deprived working-class areas,^27^ IMG (and BAME) GPs worked disproportionately in inner-city areas, ex-mining communities, and coastal towns,^30^ and GPs who qualified in Bangladesh, India, Pakistan, and Sri Lanka had more patients than average living in deprived areas.^15^

Working in more deprived areas was linked to financial, workload, and patient challenges. Remuneration for GPs working in inner-city areas was often less than that of doctors working in more suburban areas.^15^ The additional funding for GPs in more deprived areas may not be sufficient to compensate for multiple patient comorbidities, which is linked to heavier workloads.^30^ An analysis of GPs who qualified in Bangladesh, India, Pakistan, and Sri Lanka found that these GPs practised, on average, in areas with more mobile populations with an excess of mental health problems, making it more difficult to meet targets.^15^

#### Language and communication

IMG GPs had challenges communicating with native English speakers, even when they performed well on (standardised) English language tests and understood English grammar and vocabulary.^21^ IMG GPs sometimes struggled to understand idioms and accents, and used phrases translated from their native languages or intonation patterns that native speakers perceived negatively.^21,30^ According to one study, sociolinguistic errors are judged more harshly by native speakers than grammatical mistakes, meaning that IMG GPs whose first language is not English may unintentionally jeopardise their rapport with patients.^21^ IMG GPs born in South Asia described being trained in ‘*the kind of language which is spoken in England*’ as facilitating their careers and potentially shielding them from discrimination.^27^

#### Visas

Once IMG GPs have completed their GP training, most need to find a GP practice to sponsor their visa, or risk being forced to leave the country.^31^ IMG GPs can apply for an ‘Indefinite Leave to Remain’ after 5 years of residency, but GP training takes only 3 years. This problem is unique to general practice, since other specialty training takes a minimum of 5 years.^31^ A Royal College of General Practitioners (RCGP) study found that few practices were willing to ‘sponsor’ visas as the process was onerous, and that most practices only started this process once they had identified an IMG GP they wished to recruit, leading to stress and uncertainty.^31^

#### Lack of NHS-specific knowledge

Four studies^8,21,23,30^ briefly mentioned that some early career IMG GPs lacked NHS-specific knowledge, including a lack of confidence to practise independently following an induction scheme,^21^ little experience in interviewing employers and negotiating job plans,^23^ and a lack of knowledge about GP induction and refresher Schemes.^8^ One study noted that IMG GPs were not perceived to ‘*know the rules*’ and did not understand the ‘*hidden curriculum*’ for practising *‘the art of medicine*’ in the UK, which included knowledge surrounding personal interactions and clinical practice that was taken for granted (and thus likely uncodified).^30^

### Help and support available to early career IMG GPs

The studies and grey literature revealed the following range of help and support available to IMG GPs.

#### Empowerment and leadership skills training

The Next Generation GP programme empowers early career GPs by giving them the belief that they ‘*can translate insight into impact*’.^29^ The First5 initiative aims to equip early career GPs with the skills to lead the profession.^22^

#### Integration with, or representation on, national bodies

The RCGP’s First5 initiative aims to promote a ‘*sense of belonging*’ for early career GPs within the college,^22^ potentially tackling feelings of isolation.

#### Learning and development support

Early career GPs can access support for learning and development, including support for passing mandatory requirements such as revalidation, and Continuing Professional Development (CPD) via the First5 initiative.^22^ Various fellowships provide support via educational bursaries, protected learning time, and learning and development packages.^29^

#### Mentoring and coaching

Mentorship and coaching can help early career GPs ‘*get the most out of being a GP*’ and better understand the varied career opportunities available to them.^22,29^

#### Networking and peer support

Networking and peer support can help early career GPs feel less isolated, improve their confidence, and provide reassurance by understanding that others may have similar feelings and worries.^22,29^

#### Online support

Online support, such as Facebook and forums, facilitated communication and debate, and can be used to link early career GPs to more senior members of organisations.^22^

#### Advocating and lobbying for IMG GPs

Representatives from the Overseas Doctors’ Association (ODA) have held meetings with the government, influenced policy, and obtained representation in bodies such as the General Medical Council.^27^ The British Association of Physicians of Indian Origin (BAPIO) has advocated on behalf of IMG GPs.^16^

#### Facilitating cultural exchange and collaboration

‘*Twinning programmes*’ can facilitate cultural exchange, collaborations in research projects, and learning from each other’s experiences, and so on.^20^

#### Linguistic support

Language support, especially related to sociolinguistic and applied language skills (signposting, active listening, and so on), helps IMG GPs consult more effectively.^17,21,23^

#### Practical and financial

This support includes relocation packages,^14^ help with accommodation costs,^23^ and providing help with paperwork and practical support such as guidance on vaccine requirements.^17^ This helps IMG GPs understand what is expected of them.^17^

## Discussion

### Summary

This review identified the challenges facing early career IMG GPs and the help and support available to them. Many challenges are interrelated; for example, discrimination from patients or communication challenges may lead to higher rates of complaints. While there is some overlap between the challenges and the help and support identified, early career IMG GPs deal with complex challenges that may be insufficiently addressed by the help and support offered or perceived to be available. The findings of the included studies and grey literature were multifaceted, including recognition of the positive contribution early career IMG GPs make to the NHS and their ability to overcome obstacles,^25,27^while also noting that they require specific help and support.^15–19,30,30^ Eschewing this could lead to retention difficulties,^2^ especially given the increasingly competitive international market for doctors.^8^

The next phase of this project will aim to understand the extent to which early career IMG GPs are aware of the help and support offered, the reasoning behind their (non-)uptake of it, and the help and support they currently access and want to access, including support from non-NHS settings such as informal support networks and support in the countries in which they trained.

### Strengths and limitations

To the authors’ knowledge, this is the first review focusing exclusively on the challenges faced, and the help and support accessed, by early career IMG GPs in the NHS. It is timely in view of the ongoing workforce challenges in general practice, and will be used to inform a qualitative study about the challenges faced by IMG GPs.

The literature search was truncated owing to time and resource constraints. While there was no quality appraisal of the studies, they were all either peer reviewed or published by public bodies. The studies provided little information on the extent to which the help and support available to IMG GPs are accessed. The information included in Supplementary Table S4 is limited to the help and support outlined in the included studies, and therefore may not be exhaustive. Two included studies were published before 2000, but the challenge identified in these studies (geographical area of practice) was concordant with more recent studies.

### Comparison with existing literature

Many of the challenges identified parallel the literature on trainee IMG GPs, other IMG clinicians, and IMG GPs in other countries. A study on trainee IMG GPs in the UK highlighted similar challenges of cultural differences, difficulty with communication, and discrimination.^32^ A review of IMGs in the NHS emphasised challenges related to cultural differences and discrimination, as well as practical issues such as visas.^33^A review of the ‘foreign cultural paradigms’ facing IMGs highlighted similar themes about communication, discrimination, and varying levels of deference in doctor–patient relationships.^34^ The existing literature on trainee IMG GPs and other IMGs explored the psychosocial difficulties they faced in more detail than the studies in this review, such as fear, uncertainty, difficulty coping, and the psychological stress incurred by moving abroad.^32,33^

### Implications for research and practice

The challenges identified were not driven by IMG GPs’ lack of clinical knowledge. Tentative policy recommendations include the provision of more language training, including in idiomatic English. Training on conflict resolution and a patient-centred care models could help diffuse tensions in consultations, potentially reducing complaints and disciplinary proceedings. Early career IMG GPs’ work colleagues should inform themselves about the challenges IMG GPs face and consider how they could support them.

Future research could explore challenges surrounding visas, given IMG GPs’ uniquely vulnerable situation, and whether some IMG GPs’ beliefs and value systems may be at odds with the NHS’s organisational culture. Co-production methods (involving IMG GPs, colleagues, and patients) should be used to develop help and support for IMG GPs in the issues that they face.

## Data sharing

Date available on reasonable request

## Data

Data available on reasonable request.
